# Cloning and Expression Analysis of One Gamma-Glutamylcysteine Synthetase Gene (*Hbγ-ECS1*) in Latex Production in* Hevea brasiliensis*

**DOI:** 10.1155/2016/5657491

**Published:** 2016-06-23

**Authors:** Wei Fang, Luo Shi Qiao, Wu Ming, Qiu Jian, Yang Wen Feng, Gao Hong Hua, Xiao Xian Zhou

**Affiliations:** Key Laboratory of Biology and Genetic Resources of Rubber Tree, Ministry of Agriculture, Rubber Research Institute, Chinese Academy of Tropical Agricultural Sciences, Danzhou, Hainan 571737, China

## Abstract

Rubber tree is a major commercial source of natural rubber. Latex coagulation is delayed by thiols, which belong to the important type of antioxidants in laticifer submembrane, and is composed of glutathione (GSH), cysteine, and methionine. The rate-limiting enzyme, *γ*-ECS, plays an important role in regulating the biosynthesis of glutathione under any environment conditions. To understand the relation between *γ*-ECS and thiols and to correlate latex flow with one-time tapping and continuous tapping, we cloned and derived the full length of one *γ*-ECS from rubber tree latex (*Hbγ-ECS1*). According to qPCR analysis, the expression levels of* Hbγ-ECS1* were induced by tapping and Ethrel stimulation, and the expression was related to thiols content in the latex. Continuous tapping induced injury, and the expression of* HbγECS1* increased with routine tapping and Ethrel-stimulation tapping (more intensive tapping). According to expression in long-term flowing latex, the gene was related to the duration of latex flow.* HbγECS1* was expressed in* E. coli* Rosetta using pET-sumo as an expression vector and the recombinant enzyme was purified; then we achieved 0.827 U/mg specific activity and about 66 kDa molecular weight. The present study can help us understand the complex role of* Hbγ-ECS* in thiols biosynthesis, which is influenced by tapping.

## 1. Introduction

Rubber tree (*Hevea brasiliensis*) is a major commercial source of natural rubber [1]. Expelled from rubber tree's laticifer, latex contains laticifer cytoplasm, usual kinds of plastids, characteristic organelles (lutoids and special plastids, such as Frey-Wyssling particles), and rubber particles. In laticifers, the thiols content is nearly 1 mM/L [2], and it is one of the main physiological indices related to the field [3, 4]. To meet the growing demand for high quality rubber, it is important to increase the yield of rubber trees. Therefore, the duration of the latex flow must be restricted to control the latex yield. Thiols, which can delay coagulation of latex, belong to the important type of antioxidants which act in the laticifer submembrane; thiols are closely related to the duration of latex flow. Glutathione (GSH), cysteine, and methionine form thiols in the latex of* Hevea brasiliensis* [4]. In the latex, the concentration of glutathione and cysteine is about 0.72 and 0.44 mM, respectively [5]. The total thiol groups expressed in cytosol amounted to 2.2 ± 0.5 mM [6]; in the latex, the concentration of thiol-related cytosol is about 0.5–0.9 mM [4]. Thiols are primarily associated with the redox potential of latex; moreover, GSH accounts for a major proportion of thiols in latex of rubber tree.

In previous studies, it has been reported that GSH is involved in various processes, including storage and transport of reduced sulphur. Furthermore, GSH serves as an electron donor in biochemical reactions; it is released as a stress response to reactive oxygen and heavy metals; it is also involved in the detoxification of xenobiotics [7–12]. In addition, GSH influences the tolerance of abiotic stresses, such as frost, salt, and chill [13–15]. Recent studies have also elucidated how GSH becomes one of the important players in biotic stress management as it interacts with various established messengers [16]. Interestingly, GSH is synthesized by two ATP-dependent steps, which are catalyzed by the consecutive action of gamma-glutamylcysteine synthetase (*γ*-ECS); this enzyme forms gamma-glutamylcysteine (*γ*-EC) from cysteine and glutamate, and glutathione synthetase (GSHS) adds glycine to the *γ*-EC. Therefore, *γ*-ECS is regarded as a key enzyme in the biosynthesis of GSH [17–19].

In plants, gene of *γ-ECS* was first isolated from* Arabidopsis thaliana* [20]. The expression analysis showed that the transcripts of *γ-ECS* genes accumulate when plants encounter adverse environments, such as abnormal temperatures, salinity, osmotic stress, and heavy metals [21–23]. To confirm the functions of *γ-ECS*, it was isolated from* Brassica juncea* in previous study. According to this study, transgenic rice plants with the overexpression of* BrECS* can tolerate high salinity. In other words, an overexpression of* BrECS* enhances the growth, development, and yield of rice [24]. An overexpression of bacterial *γ-ECS* in the cytosol of* Populus tremula* and* Populus alba* leads to elevated levels of GSH [17]. This indicates that transgenic plants exhibit higher Cd^2+^ uptaken in their roots. In conclusion, transgenic poplars show higher tolerance to Cd [25]. In the latex of rubber trees, thiols content is one of the important physiological parameters, while *γ*-ECS is the rate-limiting enzyme for GSH synthesis [26]. However, we hardly know the underlying molecular mechanisms for the thiols content in latex. Furthermore, we still need to decipher the rubber tree's response to stimulations with different intensities. Thus, it is necessary to isolate new genes involved in the latex of rubber trees. The objective of this study is to isolate *γ-ECS* genes from rubber tree and to investigate its possible physiological functions against the thiols in the latex.

## 2. Materials and Methods

### 2.1. Materials

Latex is expelled and collected by successive tapping. Rubber tree clones Reyan8-79, Reyan7-33-97, PR107, and RRIM600 were grown in the Experimental Farm of the Chinese Academy of Tropical Agricultural Sciences, Hainan Province, China. Clones of Reyan8-79 latex samples were collected from ten-year-old virgin trees without treatment. Reyan7-33-97 was a young tapped rubber tree; for the first year, it was subjected to 2% Ethrel stimulation. It was used for collecting latex after being stimulated at different time periods (four different treatments were applied during 0, 12, 24, and 48 h). For different tapping intensity (including no tapping, routine tapping, and Ethrel stimulation), we used the clone PR107 in a thirty-year-old tree. We collected latex samples at different time periods (morning latex, afternoon latex, and next-day latex) from old rubber tree RRIM600. The samples of leaves, root, xylem, bark, and latex were collected from virgin Reyan7-33-97 trees without treatments.

### 2.2. Cloning of Two *γ-ECS* Genes

The extraction of total RNA was performed from 1 mL of fresh latex from rubber tree Reyan8-79 by the Plant RNA Mini Kit (Bioteke, China). Then, 1 *μ*g of total RNA was annealed to an oligo (dT)18 primer and reverse-transcribed at 42°C for 1 h. Thus, we obtained the first strand of complementary DNA (cDNA) using RevertAid First Strand cDNA Synthesis Kit (Fermentas, USA) according to the manual. The homologously expressed sequence tags (EST) of* Hbγ-ECS1* were selected from the latex transcriptome database, and their full length was determined by rapid amplification of cDNA ends (RACE). Four primers (3′RACE-GSP: 5′-AAACA GGGAAAGCAGAGCA-3′, 3′RACE-NUP: 5′-ACATGCACTGTCCAGGTTAA-3′/5′RACE-GSP: 5′-TCTGCTTTCCCTGTTTGAGTCCTAT-3′, and 5′RACE-NSP: 5′-CCTC TTTGGTTAGCGGTTCT-3′) were used for 3′-terminus RACE and 5′-terminus RACE, respectively. Furthermore, PCR products were purified using the Agarose Gel DNA Purification Kit Ver. 2.0 (TaKaRa, Japan) and then they were sequenced. The open reading frame (ORF) region was identified using http://www.ncbi.nlm.nih.gov/gorf/gorf.html. Thereafter, the ORF region was verified by high fidelity PCR amplification (PrimeSTAR® HS DNA Polymerase, TaKaRa, Japan).

### 2.3. Bioinformatic Analysis

From the NCBI databases, we performed homology searches with BLAST (http://www.ncbi.nlm.nih.gov/BLAST) as the default parameter. Furthermore, the full length of amino acid sequences from *γ*-ECS of six organisms was aligned by ClustalW and imported into the Molecular Evolutionary Genetics Analysis (MEGA) package version 5 [27]. Then, the neighbor-joining method was used for performing phylogenetic analysis in MEGA. Bootstrap tests were conducted by 1000 replicates; the branch lengths were proportional to phylogenetic distances.

### 2.4. Analysis of Thiols Content

The thiols content in latex was measured by dithionitrobenzoic acid (DTNB) in a colorimetric method according to the protocol described in a previous study [2].

### 2.5. Gene Expression Analysis

Gene expression levels were detected by quantitative real-time PCR (qPCR) using 18S rRNA (GenBank Accession Number: AB268099) as an internal control (18s rRNA primers: 5′-GGTCGCAAGGCTGAAACT-3′/5′-ACGGGCGGTGTGTACAAA-3′). Total RNA (1 *μ*g) was isolated from Reyan8-79, Reyan7-33-97, PR107, and RRIM600 latex. Single-stranded cDNA was prepared by 1.0 *μ*g of total RNA which was reverse-transcribed from each sample using a one-step RT-PCR kit (Fermentas, USA). The primers' sequences were as follows: 5′-GAAAGCTGTTGCAGAGG AAATG-3′/5′-TCATATCTTCCCTTGGGCATAAC-3′. The SYBR Green real-time PCR assay was carried out in a total volume of 20 *μ*L, containing 10 *μ*L of 2x SYBR Green Master Mix (Applied Biosystems), 0.2 *μ*M (each) of specific primers, and 100 ng of template cDNA. The amplification was achieved by the following PCR protocol: denaturation was carried out at 95°C for 30 s; then we performed 40 cycles of denaturation at 95°C for 5 s, annealing at 60°C for 20 s, and extension at 72°C for 20 s. During each analysis, a negative control without a cDNA template and a reference gene 18SrRNA were run to normalize the data and to evaluate the overall specificity. All the reactions were carried out in triplicate in 96-well plates of a CFX96 Real-Time PCR System (Bio-rad). The PCR products were analyzed using 2.0% agarose gels, which were stained with ethidium bromide to ensure that their sizes were within acceptable limits. The expression levels are presented as a ratio relative to the control sample, which was set as 1.

### 2.6. Statistical Analysis

All the experiments were repeated at least three times using three biological replicates and every replicate contained at least five trees. The significant differences between data sets were evaluated by Student's *t*-test (5% significance, *P* < 0.05). The calculations were carried out by Microsoft Excel software.

### 2.7. Expression of Recombinant* Hbγ-ECS1* in* E. coli* Rosetta

The purified double-restricted PCR product (primer: 5′-gactATGGCGCTT GTTTCTCAGGCAGGC-3′; 5′-ggcctTTAGTACAGTAGTTCCTCAAAAAC-3′) of* Hbγ-ECS1* was ligated within pET-sumo using T4 DNA ligase in its specific buffer. Ligation mixture transfected with competent cells* E. coli* DH5*α* and positive clones were confirmed by PCR and gene sequencing. The recombinant plasmid DNA was extracted and transformed* E. coli* Rosetta. The transformed* Rosetta* cells were grown in a 100 mL LB medium to an optical density of 0.5–0.7 at 37°C. The cells were induced by 0.5 mM Isopropyl *β*-D-1-thiogalactopyranoside (IPTG) and were harvested by centrifugation (8000 ×g 180 s). Pellet obtained was resuspended in NTA-0 buffer and disrupted by ultrasonic wave. Expression of* Hbγ-ECS1* was determined in both intracellular and extracellular fractions by 12% SDS-PAGE and was visualized after staining with a Coomassie Brilliant dye.

### 2.8. Fermentation, Purification of Inclusion Body, and Renaturation of Protein

100 mL bacterium suspension was cultivated and centrifuged. Pellet was resuspended and disrupted by ultrasonic wave. The inclusion body was vibrated and resuspended until dissolved. Dissolved body was centrifuged and the supernatant was denatured protein. Purified protein was added in renaturation buffer (0.2% PEG 4000, 1 mM GSSG, and 2 mMh GSSH) for 12 h at 4°C and dialyzed in TE buffer for 3 days. The renaturation protein estimation was performed by the method of Bradford.

### 2.9. Enzyme Activity

The purified renaturation protein was assayed according to Rüegsegger and Brunold [28, 29]. For *γ*-ECS activity, the reaction was started by addition of the enzyme extract (140 *μ*L) to give 500 *μ*L assay mix containing 100 mM Hepes (pH 8.0), 50 mM MgCl_2_, 20 mM glutamate, 1 mM cysteine, 5 mM ATP, 5 mM phosphoenolpyruvate, 5 mM DTT, and 10 U mL pyruvate kinase. The reaction mixture was incubated at 37°C for 45 min, and the reaction was stopped by addition of 100 *μ*L of 50% TCA. The mixture was centrifuged, and the supernatant was used for estimation of phosphate content by the phosphomolybdate method.

## 3. Results

### 3.1. Bioinformatic Analysis

**Figure 1:**
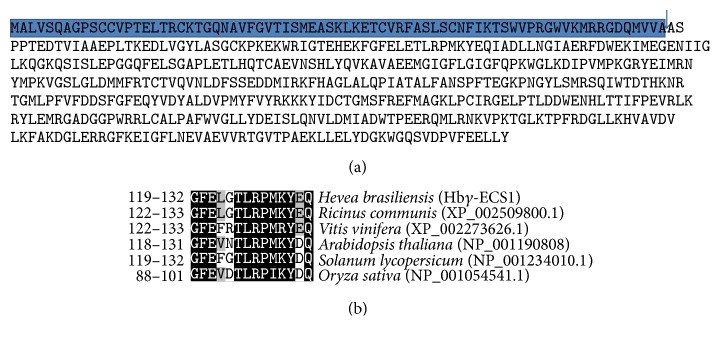
Analysis of deduced amino acid sequence: (a) the deduced amino acid sequence of Hb*γ*-ECS1, showing the 74 amino acids in a long transit peptide of chloroplast, and its putative cleavage site (VAA). (b) Comparison of the deduced amino acid sequence of* Hevea brasiliensis* and other plant sources with a putatively oxidized GSH binding site.

**Figure 2:**
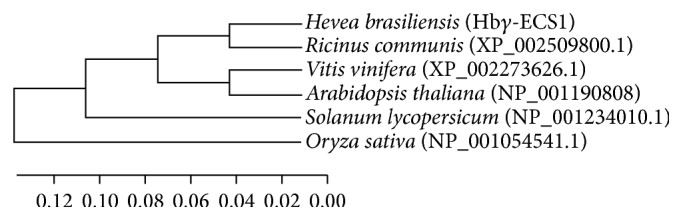
Phylogenetic analysis of Hb*γ*-ECS1, Hb*γ*-ECS2, and five other plants. The Latin name of the species and NCBI accession number are provided. The phylogenetic tree is based on the genetic distances of protein sequences, and it was generated by MEGA5.01 software using ClustalW for the alignment. We used a neighbor-joining algorithm with a total of 1000 bootstrap replicates.

A new cDNA of *γ*-ECS homologous gene,* Hbγ-ECS1*, was isolated from* Hevea brasiliensis*. The obtained sequence included the translation start site (ATG) along with 223 bp of 5′UTR region and 168 bp of 3′UTR region, which were downstream to the stop codon (TAA). The complete ORF was composed of 1572 bp, encoding a polypeptide of 523 amino acids. The TargetP and the ChloroP software predicted the presence of a putative transit peptide of 74 amino acids in the* Hbγ-ECS1* putative protein sequence of chloroplast. It also detected the presence of conserved cleavage site Val AlaAla (VAA) (Figure 1(a)). The putative Hb*γ*-ECS1 protein displayed 90%, 82%, 78%, 81%, and 71% sequence similarity with *γ*-ECS protein sequence, which was isolated from* Ricinus communis*,* Vitis vinifera*,* Arabidopsis thaliana*,* Solanumly copersicum*, and* Oryza sativa*, respectively (Figure 2).

Upon multiple alignment of all the above six sequences, we observed that, barring the exception of transit peptide sequence, the remaining showed high similarity in their sequences. Notably, the transit peptide cleavage site in the plastids also had similar sequences. As shown in Figure 1(b), the putatively oxidized GSH binding site was also conserved [29].

### 3.2. Gene Expression

#### 3.2.1. Tissue-Specific Expression

**Figure 3:**
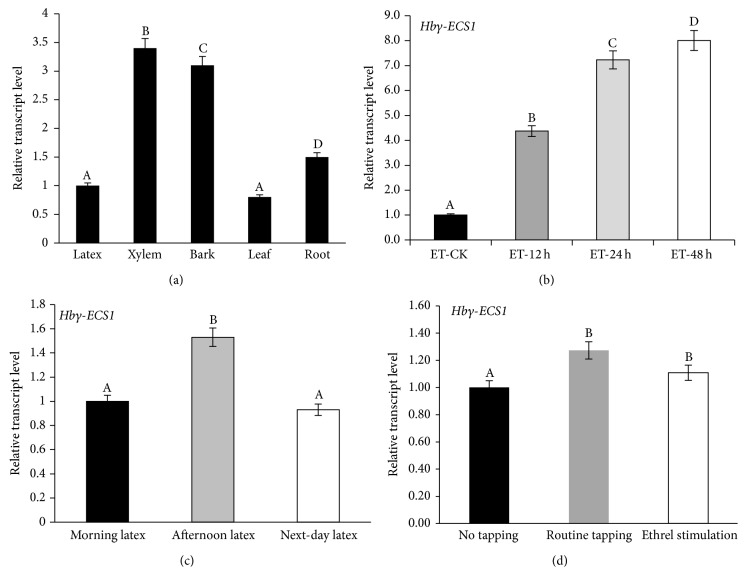
Expression analysis of two genes. (a) Expression profile of Hb*γ*-ECS1 in different tissues. (b) Expression patterns and relative mRNA levels of* Hbγ-ECS1* at different tapping times after Ethrel stimulation. (c) Expression patterns and relative mRNA levels of* Hbγ-ECS1* and* Hbγ-ECS2* in long-term flowing latex of RRIM600. The long-term flowing latex was divided into the following flow times: morning latex, afternoon latex, and next-day latex. (d) Expression patterns and relative mRNA levels of* Hbγ-ECS1* and* Hbγ-ECS2* after being subjected to three months of every tapping intensity in PR107 (this figure expressed the difference in three months). Tapping system: half spiral tapping every 3 days along with Ethrel stimulation (15 d). Quantitative RT-PCR analysis was carried out to determine the expression levels. The 18SrRNA (GenBank Accession Number: AB268099) was used as the control. Data are expressed as mean ± SD for three individual experiments (*n* = 3).

Real-time PCR reactions were carried out to determine the expression of* Hbγ-ECS1* in the five tissues (latex, xylem, bark, leaf, and root). The results showed that* Hbγ-ECS1* had a low expression level in the latex and leaf tissues, but it was highly expressed in the xylem and bark tissues (Figure 3(a)).

#### 3.2.2. Expression Level Stimulated by Ethrel and Relation between Gene and Thiols Content in the Latex

Ethrel (2-chloroethylphosphonic acid) was used as a stimulation to increase latex production and reduce labor productivity. The expression level of* Hbγ-ECS1* was promoted 4 times after stimulation for 12 h and increased 8 times for 48 h. Ethrel stimulation significantly regulated the expression of* Hbγ-ECS1* (Figure 3(b)).

Thiol is one of the main physiological indices which are related to latex field. The physiological parameters (sugar content, inorganic phosphorus, etc.) of latex were comprehensively analyzed to find out why thiols content decreases within 24 h Ethrel stimulation after the metabolism of laticifers system was subjected to vigorous stimulation. We proposed that the consumption of thiols was more active than synthesis [27]. However, the thiols content increased due to the addition of thiols in 48 h Ethrel stimulation. It can be speculated that, at the initial stages, the high level of expression of* Hbγ-ECS1* complements the thiols content that was consumed previously by vigorous metabolism. This indicates that the gene regulated by Ethrel is related to the thiols content in the latex of rubber trees.

#### 3.2.3. Gene Expression Level in Long-Term Flow Latex

The long-term flow of latex was the result of intensive tapping [30]. Based on ethylene gas-stimulation, long-term flowing latex in old-age RRIM600 rubber tree began from the first day of morning tapping until the morning of the next day, so the time periods of latex flow were divided into morning latex, afternoon latex, and next-day latex. The expression level of* Hbγ-ECS1* was higher in the afternoon latex than at the other times (Figure 3(c)). It can be speculated that thiols were consumed during the course of latex expulsion and the increasing expression of related genes complemented the reductive thiols.

#### 3.2.4. Expression Level at Different Tapping Intensity

The degrees of three tapping intensities varied from weak to strong as follows: no tapping, routine tapping, and Ethrel-stimulation tapping. The degree of tapping intensity represents the level of rubber tree injury that was subjected to continuous tapping. Compared with the control (no tapping), the expression of* Hbγ-ECS1* increased significantly in the case of routine tapping and Ethrel-stimulation tapping for three months (Figure 3(d)).

### 3.3. Purification of Recombinant Enzyme

**Figure 4:**
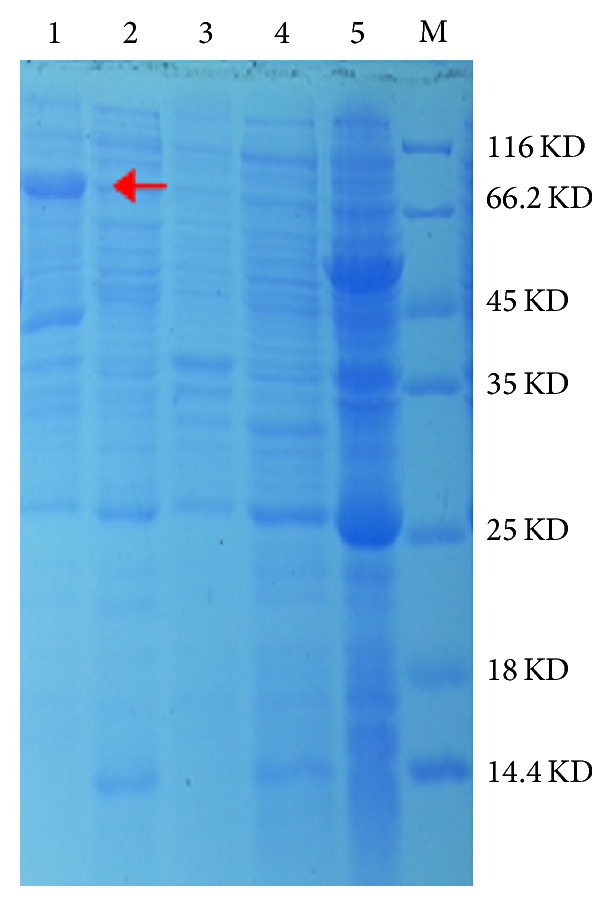
SDS-PAGE analysis of cloned* Hbγ-ECS1* expression. Partially purified recombinant* Hbγ-ECS1*, noninduced strain Rosetta/pET-sumo-*Hbγ*-*ECS1*, induced empty vector strain Rosetta/pET-sumo, wild* E. coli* Rosetta, cell supernatant of induced strain Rosetta/pET-sumo-*Hbγ-ECS1*, and protein ladder are present in lanes 1, 2, 3, 4, 5, and M, respectively.

The purified fraction of* Hbγ-ECS1* was examined by running SDS-PAGE. Distinct protein band of 66 kDa was perceived as purified* Hbγ-ECS1*. No band at this position was observed in the controls (noninduced strain Rosetta/pET-sumo-*Hbγ-ECS1*, induced empty vector strain Rosetta/pET-sumo, wild* E. coli* Rosetta, and cell supernatant of induced strain Rosetta/pET-sumo-*Hbγ-ECS1*) (Figure 4).

### 3.4. Enzyme Activity

It was observed that the concentration of renaturation protein was 0.3 mg/mL. One enzyme activity unit was defined as 1 *μ*mol inorganic phosphorus produced by ATPase decomposing ATP per hour per mg tissue protein. The enzyme activity calculation formula is as follows:

(1) γ-ECS  enzyme  activity  U/mg=measured  OD−control  ODStandard  OD−blank  OD×Concentration  of  standard  sample×60 min6 min÷Concentration  of  sample  protein.

According to the measured OD, the mean result of enzyme activity was 0.827 U/mg.

## 4. Discussion

More reactive oxygen species (ROS) were generated by tapping [31], Ethrel stimulation [32], and latex flowing. The release of reactive oxygen is claimed to be responsible for the peroxidative degradation of the lutoid membrane. Lutoid is a special organelle in latex, in which hevein proteins rich in cysteine occupied 70% in lutoid whole proteins and hydrophobic groups with reduction activity exposed to protein surface. Hydrophobic groups linked N-acetyl glucose on rubber particles membrane under oxidation, which caused gathering of rubber particle and stopping of latex flowing [33]. Thiols, eliminating ROS through redox reaction, are important antioxidants to laticifer submembrane and beneficial to latex stability, prolonging flowing time and increasing production. GSH accounts for a major proportion of thiols in latex of rubber tree.

In higher plants, GSH is associated with protective mechanisms because it has multifaceted functions inside plant cells [14, 34–36]. Our results focused on the cloning and sequence analysis of *γ-ECS*, a rate-limiting enzyme that is associated with GSH biosynthesis. Thus, we comprehensively investigated the regulation of mRNA expression levels in different clones of rubber trees that were subjected to Ethrel stress. We also determined the long-term flow of latex that is induced by continuous tapping. In* Arabidopsis thaliana*, a single copy of *γ-ECS* gene is present; in the* Oryza sativa* genome, two *γ-ECS* genes are present. In this case, we isolated one *γ-ECS* from latex of rubber tree and denoted it as* Hbγ-ECS1*. An identity analysis showed that Hb*γ*-ECS1 is identical to *γ*-ECSs found in other plants. Hb*γ*-ECS1 showed a similarity of 78% and 71% with *γ*-ECS of* Arabidopsis* and rice, respectively. The putative target peptide cleavage sequences are highly conserved, but the target itself is not conserved. As Hb*γ*-ECS1 has sequences that are highly similar to those of other plants, we conclude that the *γ*-ECS genes are highly conserved and their functions were important for the protein. This finding was in full agreement with the results of earlier studies [29].

When plants were challenged by heavy metals, salt, herbicides, hormones, extreme temperatures, and osmotic stresses, the expression levels of *γ-ECS* genes were stimulated [22, 37]. Owing to Ethrel stimulation, the latex yield increases up to 1.5–2.0-fold in rubber tree. Ethrel can improve latex yield, mainly by prolonging latex flow [4]. “Long-term flow” is a distinct feature of rubber tree long-term flowing latex (LFL); it has a longer flowing time than the normal flowing latex (NFL), whose flowing time is less than 6 h. On the one hand, the long duration of flowing time increases latex yield per tapping; on the other hand, rubber tree expends a lot of metabolic energy in case of long-term flow, so rubber plants take longer time for their renewal under such circumstances [30]. Our result indicated that Ethrel and long-term flow could enhance the mRNA transcription of* HbγECS1* in the latex. Consequently, as more thiols were synthesized in the latex under the stimulating conditions, the GSH-synthesizing capacity was also enhanced; thereby GSH levels were elevated [23]. Indeed, more GSH was detected in the latex when rubber tree was subjected to Ethrel stimulation. These results indicated that the regulatory mechanisms of* Hbγ-ECS1* in* Hevea brasiliensis* are similar to those encountered in other plants.* HbγECS1* was more sensitive to Ethrel and increased significantly according to extending of Ethrel stimulation. However, owing to Ethrel and LFL, the latex production was higher than that witnessed in NFL. Hence, the expression of this gene is related to latex production.

Owing to Ethrel stimulation and long-term flow, the ephemeral stress response of* HbγECS1* was elicited in one tapping. Tapping means a kind of continuous injury to rubber tree. The expression level of* HbγECS1* varied with different tapping intensities, showing a durable reaction to tapping. Obviously, the expression levels of short-term and long-term tapping were not similar. There was an increase in the transcriptional levels of* HbγECS1* after the rubber tree was subjected to three-month routine tapping, but not significantly in Ethrel-stimulation tapping. This indicates that there was a feedback regulation mechanism according to the more tapping intensity; however, researchers had elucidated the role of feedback inhibition by GSH in *γECS* by a series of experiments. The injury induced by tapping and Ethrel stimulation could induce the generation of reactive oxygen in rubber tree latex [38–40]. The release of reactive oxygen leads to the peroxidative degradation of lutoid membrane. Moreover, thiol was one of the substances scavenging reactive oxygen, so it was closely related to the duration of latex flow. The expression of* Hbγ-ECS1* was regulated by the tapping intensity on rubber tree.* HbγECS1* was expressed in* E. coli* Rosetta using pET-sumo as an expression vector and the recombinant enzyme was tested for its ability catalyzing *γ*-EC biosynthesis from cysteine and glutamate. The molecular weight was about 66 kDa and frees up 7 KD comparing to anticipation because of his-tags.

On the basis of the information gathered in the present study, it was suggested that* Hbγ-ECS1* was a potential candidate that may be utilized in rubber tree genetic breeding for prolonging latex flowing time and increasing resistance.

## Abbreviations

*γ*-ECS: Gamma-glutamylcysteine synthetase
GSH: Glutathione
GSHS: Glutathione synthetase
*γ*-EC: Gamma-glutamylcysteine
EST: Expressed sequence tags
RACE: Rapid amplification of cDNA ends
ORF: Open reading frame
qPCR: Quantitative real-time PCR
DTNB: Dithionitrobenzoic acid
MEGA: Molecular Evolutionary Genetics Analysis
TPD: Tapping panel dryness.
